# Supplementary material for: Nano-Pulse Treatment Overcomes the Immunosuppressive Tumor Microenvironment to Elicit In Situ Vaccination Protection against Breast Cancer
Source: Vaccines (Basel). 2024 Jun 7;12(6):633. doi: 10.3390/vaccines12060633 (PMC11209453; doi:10.3390/vaccines12060633)
Supplement: Supplementary file 1 [file vaccines-12-00633-s001.zip › vaccines-3012667-supplementary.pdf]

Supplementary Material

Nano pulse treatment overcomes immunosuppressive tumor microenvironment to elicit in situ vaccination protection against breast cancer

Anthony Nanajian[1,2], Megan Scott[1], Niculina I. Burcus[1], Brittney L. Ruedlinger[1], Edwin A. Oshin[1,3], Chunqi Jiang[1,3], Stephen J. Beebe[1] and Siqi Guo[1*]

[1] Frank Reidy Research Center for Bioelectrics, Old Dominion University. Norfolk, VA, USA
[2] Department of Biological Sciences, Old Dominion University. Norfolk, VA, USA
[3] Department of Electrical & Computer Engineering, Old Dominion University, Norfolk, Virginia, USA,
*Correspondence to:
Siqi Guo, Phone: 757-683-7021. Fax: 757-451-1010. Email: s2guo@odu.edu

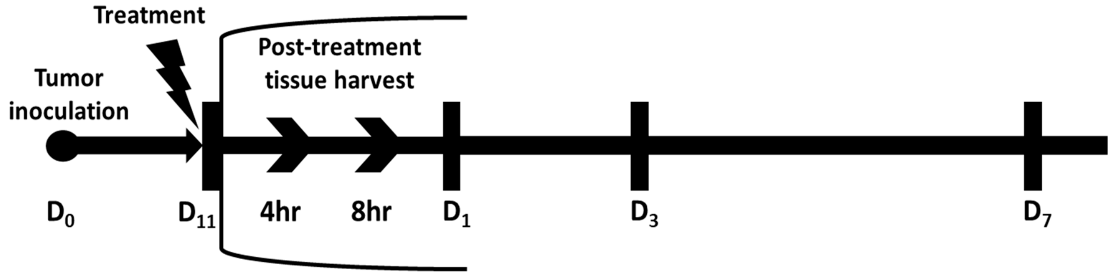

Supplementary Figure S1.

In vivo NPT experimental design. 1x10^6 4T1-luc cells were injected subcutaneously into the posterior part of the mammary fat pad. The control group (n=4) received the tumor inoculation only. The remaining mice underwent NPT (100 ns pulses, 50 kV/cm, 1-3 Hz, 1000 pulses) on Day 11 following tumor inoculation. The treated mice were euthanized 4 hours (4hr), 8 hours (8hr) (for local tissues only), and on Day 1 (D1), Day 3 (D3), and Day 7 (D7) post-NPT. Their tumor tissues, tumor-draining lymph nodes (dLN), blood and spleens were harvested. Control tissues were obtained from mice with untreated tumors. Single cell suspensions from each tissue were prepared.

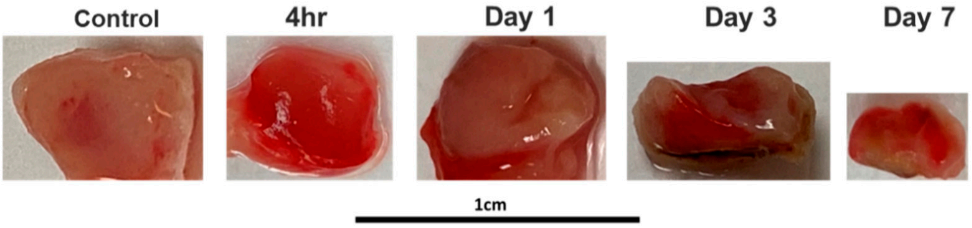

Supplementary Figure S2.

Resected 4t1-luc solid tumor samples can be seen rapidly deteriorating in size and morphology following NPT.

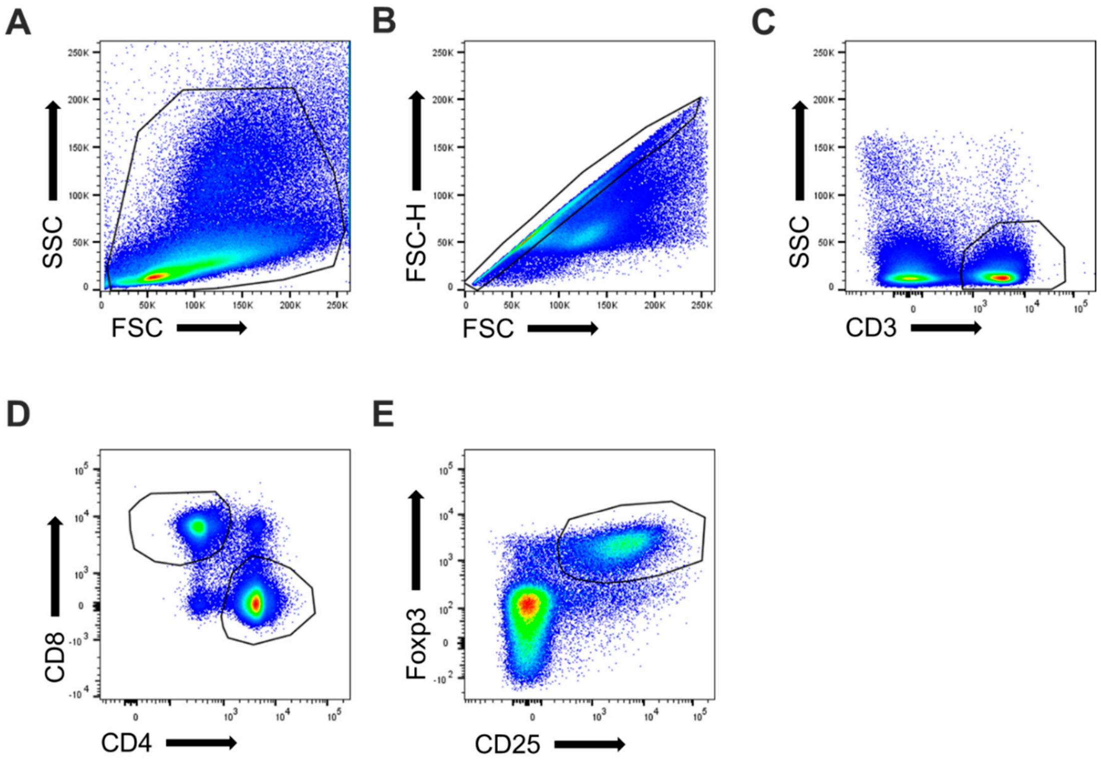

Supplementary Figure S3.

CD25⁺Foxp3⁺ Treg gating strategy. **A-E**, Summary flow plots representing the Treg gating strategy. **A**, Single cell suspensions from the dLN of 4t1-luc tumor-bearing mice were gated on the forward scatter (FSC) vs side scatter (SSC) dot plot to select lymphocytes. **B**, Lymphocytes were then gated on the FSC-Height vs FSC axis to eliminate doublets. **C** and **D**, CD3 cells were selected (**C**) and gated on the CD4 vs CD8 axis to separate CD4-single positive and CD8-single positive T cells (**D**). **E**, CD4-single positive T cells were then gated on the CD25 vs Foxp3 axis to select CD25⁺Foxp3⁺ Tregs.

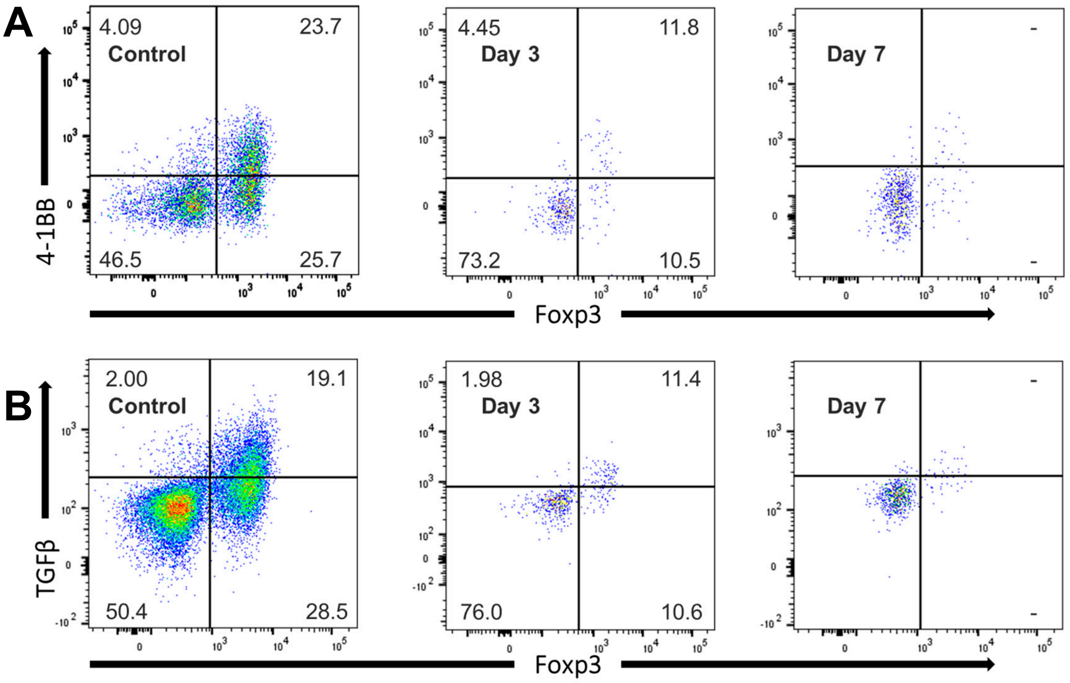

Supplementary Figure S4.

NPT severely reduces intratumoral Tregs making their activation marker expression unquantifiable post-treatment. **A** and **B**, Flow plots representing Foxp3 co-expression with 4-1BB (**A**) and TGFβ (**B**) among CD4$^+$ TILS in untreated tumors and on Day 3 and Day 7 post-NPT. N=4 per group.

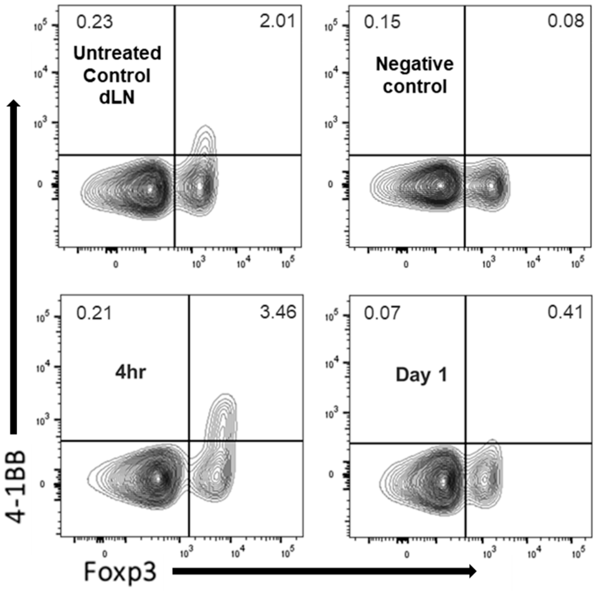

Supplementary Figure S5.

NPT-induced changes in 4-1BB and Foxp3 co-expression among CD4$^+$ T cells in the dLN. Flow plots represent 4-1BB and Foxp3 analysis among CD4$^+$ T cells in the dLN of untreated tumor-bearing mice (Untreated control), tumor-naïve mice (Negative control) and treated mice from the 4-hour and Day 1 post-NPT timepoints. N=4 per group.

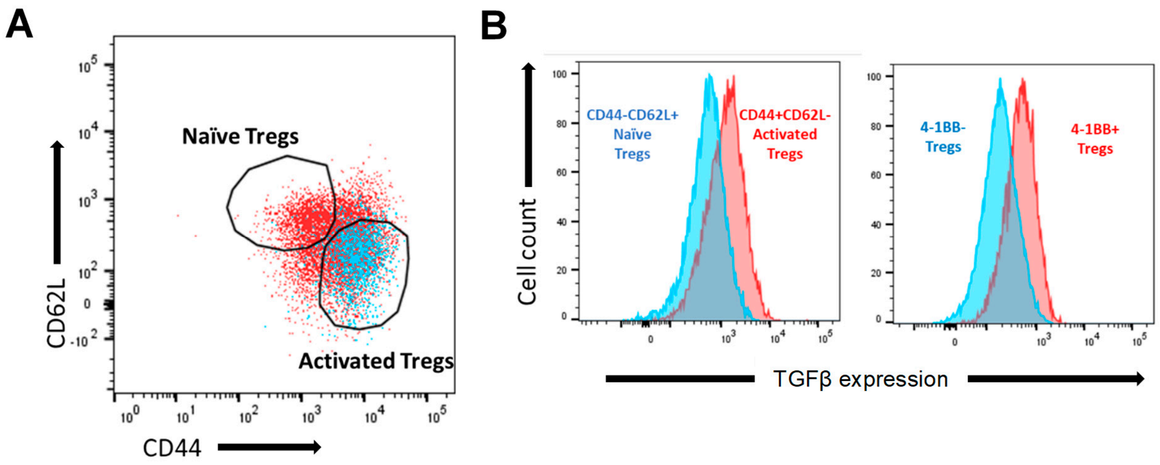

Supplementary Figure S6.

Comparing phenotypic characteristics among different Treg subsets in the untreated tumor dLN. **A,** Flow plots represent CD44/CD62L expression among the total Treg population (red) and 4-1BB^{+} subgate (blue) in the dLN of untreated tumor-bearing mice. **B,** TGFβ expression among naïve vs activated Tregs (left) and 4-1BB^{+} vs 4-1BB^{-} Tregs (right).

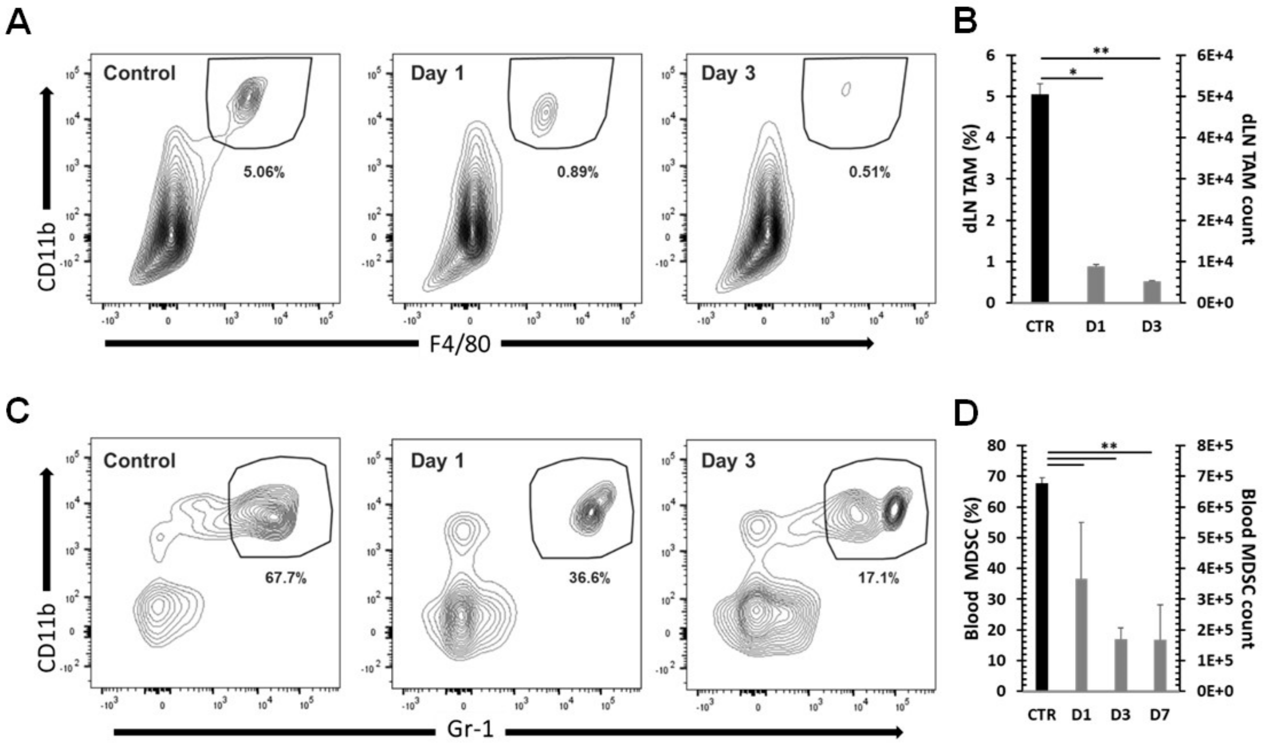

Supplementary Figure S7.

MDSCs and TAMs are decreased following NPT. F4/80^{+} CD11b^{+} TAMs in the dLN (**A** and **B**) and Gr-1^{+} CD11b^{+} MDSCs in the blood (**C** and **D**) are represented as a percentage among total single events in the summary flow plots (**A** and **C**) and quantitative bar graphs (**B** and **D**). A standardized cell count is represented for TAMs (**B**) and MDSCs (**C**). N=4 per group. Error bars, SD. ** $p < 0.01$ and * $p < 0.05$ determined by one-way ANOVA.
